# Maternal satisfaction with organized perinatal care in Serbian public hospitals

**DOI:** 10.1186/1471-2393-14-14

**Published:** 2014-01-13

**Authors:** Bojana Matejić, Milena Šantrić Milićević, Vladimir Vasić, Bosiljka Djikanović

**Affiliations:** 1 Institute of Social Medicine, Faculty of Medicine, University of Belgrade, Dr Subotica 15, 11 000 Belgrade, Serbia; 2 Department of Statistics and Mathematics, Faculty of Economics, University of Belgrade, Belgrade, Serbia

## Abstract

**Background:**

Understanding the experiences and expectations of women across the continuum of antenatal, perinatal, and postnatal care is important to assess the quality of maternal care and to determine problematic areas which could be improved. The objective of this study was to identify the factors associated with maternal satisfaction with hospital-based perinatal care in Serbia.

**Methods:**

Our survey was conducted from January 2009 to January 2010 using a 28-item, self-administered questionnaire. The sample consisted of 50% of women who expected childbirths during the study period from all 76 public institutions with obstetric departments in Serbia. The following three composite outcome variables were constructed: satisfaction with technical and professional aspects of care; communication and interpersonal aspects of care; and environmental factors.

**Results:**

We analyzed 34,431 completed questionnaires (84.2% of the study sample). The highest and lowest average satisfaction scores (4.43 and 3.25, respectively) referred to the overall participation of midwives during delivery and the quality of food served in the hospital, respectively. Younger mothers and multiparas were less concerned with the environmental conditions (OR = 0.55, p = 0.006; OR = 1.82, p = 0.004). Final model indicated that mothers informed of patients’ rights, pregnancy and delivery through the Maternal Counseling Service were more likely to be satisfied with all three outcome variables. The highest value of the Pearson’s coefficient of correlation was between the overall satisfaction score and satisfaction with communication and interpersonal aspects of care.

**Conclusions:**

Our study illuminated the importance of interpersonal aspects of care and education for maternal satisfaction. Improvement of the environmental conditions in hospitals, the WHO program, Baby-friendly Hospital, and above all providing all pregnant women with antenatal education, are recommendations which would more strongly affect the perceptions of quality and satisfaction with perinatal care in Serbian public hospitals by women.

## Background

In an attempt to assess and improve the quality of the health care system, particularly over the last two decades, increasing importance has been given to the opinions, expectations and experiences of the users of the health care system. Patient satisfaction is a subjective and dynamic perception of the extent to which the expected health care is received [1]. In addition, patient satisfaction is a reflection of the patient’s judgment of different domains of health care, including technical, interpersonal, and organizational aspects [2]. The majority of published accounts indicate that satisfaction with different aspects of received health care improves health outcomes, continuity of care, compliance, and the relationship with the provider [3,4]. Understanding the experience of a patient with respect to health care is important to obtain valid information about the quality of care and to identify problematic areas which could be improved.

Across the continuum of antenatal, perinatal, and postnatal care, the assessment of maternal satisfaction has primarily focused on the following: availability of services; physical environment; hygiene and accommodation conditions; organization of work; interpersonal relationships with healthcare professionals; and the expertise and competence of healthcare professionals. Satisfaction with the aforementioned aspects of care is strongly influenced and shaped by socio-demographic characteristics of women (the level of education, age, marital status, and economic status), the number of personal factors (values, attitudes, threshold of pain, health literacy, and personal support), as well as the sense of security and perceived control and expectations formed on the basis of previous experiences and outcomes of previous pregnancies and births [5-9]. Focusing on the significance of expectations, as did Janzen et al. [10], maternal satisfaction with prenatal care could be argued to be an experience that results from a subjective assessment of what women expected was supposed to happen in relation to delivery and what really happened. Having in mind the plethora of influences, maternal satisfaction is the outcome of a broad range of objective circumstances, both clinical and technical, but also of the many factors which are subjective by nature [11,12].

The World Health Organization recommends monitoring and evaluation of maternal satisfaction in public health care sectors - to improve the quality and efficiency of health care during pregnancy, childbirth, and the puerperium [13]. The Ministry of Health of Serbia initiated research on maternal satisfaction in public healthcare institutions throughout Serbia in December 2008. Inpatient maternal care is provided through the network of public institutions, including university hospitals- clinics and institutes of gynecology and obstetrics, maternity departments in general hospitals, and specialized small unit maternity departments within primary health care institutions^a^. All services at these institutions are provided free of charge. According to the official state report, 99.6% of all deliveries took place in the public health institutions [14]. The study period was preceded by a rising public interest in the quality of care in public maternity hospitals. The increased media attention focused on the statements and reactions of mothers regarding the inappropriate communication and behavior of the medical professionals, outdated hospital protocols and additional out-of-pocket payments for the services which were already covered by insurance. The opening of this “Pandora’s box” was followed by a blog on the Internet with the aim of collecting and publishing women’s experiences from the maternity hospitals, which later became a full website. The social engagement of women ultimately was institutionalized as a non-governmental organization. The assessment of maternal satisfaction was planned to be conducted according to the governmental strategy for continuous quality improvement and patient safety [15], but certainly, it was partly driven by the aforementioned activities of the public initiative. Furthermore, a series of state and public activities were conducted to promote the protection and realization of patients’ rights in the Republic of Serbia. At first, the promotion of principles of health care laws and regulations, then the introduction of patients’ rights guardians in all health facilities, the promotion of patients’ rights at national and international conferences, mass media campaigns to inform patients (“You’re right” and “Health is spread with a smile”), and the projects for cooperation between state and patient organizations (for example, in projects such as “Protection of patients’ rights at the local level”).

The aim of our study was to assess the multidimensional nature of patient satisfaction and to identify the factors associated with maternal satisfaction with hospital-based perinatal care in Serbia.

## Methods

### Study design, settings and participants

This paper is based on the secondary data analysis of an existing dataset which had previously been collected in the large cross-sectional study of women’s satisfaction with perinatal care. The study was organized and conducted by The Ministry of Health of Republic of Serbia in cooperation with the Institute of Public Health of Serbia. The study population comprised of all women who recently had a birth during the study period (September 2009 to January 2010), in one of the 76 public institutions with obstetric departments in Serbia. The midwife home-care service from each health care institution provided a questionnaire to women during the first home visit, 5–7 days after delivery. All respondents received clear, written information about the nature and the purpose of the survey, and the identity of the institutions which initiated, organized and conducted the survey. The women could withdraw their consent at any time by refusing to cooperate. The questionnaires were accompanied by an envelope for return. The women had one day to answer the questionnaire in the privacy of their homes, and to enclose it in the prepared envelope, which the midwives collected next day during the second home visit. The design of this survey was approved by the ethics committee of The Ministry of Health of Republic of Serbia.

### Data collection

The study instrument was self-administered questionnaire, designed, validated and pretested for the purpose of the large cross-sectional study of women’s satisfaction with perinatal care in Serbia. The questionnaire was developed by a team of public health professionals who are experienced in conducting surveys, with cooperation of two obstetricians. The first version of questionnaire was piloted on 10 women who recently gave birth. After the clarification of certain response categories, the final form of questionnaire was tested for face and content validity on another 10 women. This analysis focused on whether the concepts and items comprising the questionnaire were relevant, appropriate and well understood by the women in the way intended by the developers [16].

The first section of the study instrument was comprised of independent (explanatory) variables. The questions addressed the mother’s age, level of education, and parity (primiparous or multiparous). Mode of delivery was dichotomized: normal vaginal delivery (spontaneous vaginal birth regardless of the anesthesia) or caesarean. This was followed by variables in which women answered to what extent they were informed of their guaranteed patients’ rights in Serbian health care system: to access health care, to be informed, to have freedom of choice, privacy and confidentiality of information, independent decision-making and consent, access to medical documentation, consent to medical trials, to complain, damage compensation, and public information. The health services ought to guarantee the exercise of these rights, so we asked the respondents if they knew the standard procedures to formalize their complaint if they had suffered a violation of these guaranteed rights. Control of pregnancy is typically performed by a doctor gynecologist from the institution of primary health care, through dispensaries for women, counseling through Maternal Counseling Service and finally home visits. One question addressed received antenatal education through the Maternal Counseling Service, regarding the care, nutrition and other lifestyles during pregnancy, childbirth, and newborn care. That service is provided in all institutions of primary health care and it is available to all pregnant women. Two open-ended questions addressed aspects that the women identified during the hospital stay as being particularly satisfactory and the aspects that should be changed and improved. All answers from the open-ended questions were transferred into a separate file, read through several times by two researchers, sorted, and analyzed according to the technique of the qualitative content analysis [17].

Maternal satisfaction was assessed with a nineteen-item instrument which reported good internal consistency (Cronbach’s α = 0.787). The details of questions on different aspects of care received during their stay in the maternity wards are provided in the Additional file 1. All responses were marked using a 5-point Likert scale. In order to address the multidimensional nature of maternal satisfaction, we constructed three composite outcome variables, as follows: technical and professional aspects of care; communication and interpersonal aspects of care; and environmental factors. Satisfaction with one of the aspects was defined as the proportion of mothers who had chosen a mark of 4 or 5 with all the variables under this aspect of care.

### Data analysis

Data collection and analysis were performed using IBM SPSS Statistics 19. The univariate association between maternal satisfaction and independent variables was assessed by unadjusted odds ratios (ORs) with 95% confidence intervals (95% CIs). The independent associations between maternal satisfaction and relevant independent variables among variables were tested by a multiple logistic regression model in a stepwise backward manner. The variables with a p < 0.05 were retained in the final model.

## Results

### General characteristics of the sample

We collected 34,431 completed questionnaires (84.2% of the study sample). The mean age of the participants was 27.7 years (range, 15–52 years). The characteristics of the study population are presented in Table 1. The proportion of mothers delivered in clinics and clinical centers was highest (65.3%), compared with general hospitals and maternity departments of primary health care institutions. Of all mothers, 51.2% presented to a hospital for the first childbirth. More than 23% of deliveries were done by caesarean section. Approximately 58% of women were not or were only partly informed about patients’ rights and nearly 50% of mothers were not sufficiently informed about the pregnancy and delivery through the Maternal Counseling Service.

**Table 1:** Study population sociodemographic and health care-related characteristics

**Variables**	**N**	**%**
Age (years)		
<25	9294	26.9
≥25	25137	73.1
Education		
Low level education*	24457	74
Medium and high education**	8589	26
Parity		
Primiparity	16625	51.2
Multiparity	15860	48.8
Mode of delivery		
Vaginal without epidural anesthesia	23631	71.5
Vaginal with epidural anesthesia	1644	5.0
Caesarean	7785	23.5
Institution		
Primary health care	10705	32.5
General hospital	730	2.2
Clinics and clinical centers	21508	65.3
Mother informed of the patients’ rights		
Yes	14256	42.3
No or partly	19450	57.7
Mother informed where she can file a complaint for violation of the patients’ rights		
Yes	17771	52.9
No or partly	15824	47.1
Mother informed about the pregnancy and delivery through the Maternal Counseling Service		
Yes	18284	55.1
No or partly	14895	44.9

*No education, incomplete primary education, complete compulsory primary education and lower secondary education; **Upper secondary education, college education and university education.

### Dimensions of maternal satisfaction

The individual items that assessed maternal satisfaction are grouped in three scales which showed good internal consistency, as follows: the scale of environmental factors (Cronbach’s α = 0.817); the scale of communication and interpersonal aspects of care (Cronbach’s α = 0.777); and the scale of technical and professional aspects of health care (Cronbach’s α = 0.893). The average satisfaction score with individual items, as well as the percentages of satisfied mothers in relation to individual characteristics, are presented in Table 2.

**Table 2:** Proportion of mothers satisfied with three aspects of maternal care and median satisfaction scores

**Scale**	**Items**	**Median satisfaction score *_ *** **X ± SD**	**Percentage of mothers who gave mark 5 or 4**
Environmental factors	Cleanliness in the ward and the frequency of changing the nightgowns and bed sheets	3.51(1.34)	54.7
	Sanitary facilities (hygiene and equipment)	3.28 (1.39)	48.4
	Room comfort in the ward	3.75 (1.30)	63.2
	Quality of served food	3.25 (1.37)	46.1
Technical and professional aspects of care	Treatment during the preparation for childbirth	4.29 (1.07)	81.0
	Obstetrician informed mother with the plan of delivery, upcoming procedures and interventions and asked for her consent	4.05 (1.32)	73.2
	Obstetrician devoted sufficient time, provided necessary information and answered mother’s questions	4.07 (1.27)	73.5
	Midwife devoted sufficient time, provided necessary information and answered mother’s questions	4.25 (1.14)	79.0
	Neonatologist devoted sufficient time, provided necessary information and answered mother’s questions	4.11 (1.18)	74.8
	Pediatric nurse devoted sufficient time, provided necessary information and answered mother’s questions	4.07 (1.17)	73.1
	Overall participation of midwives during delivery	4.43 (1.02)	84.7
	Procedures after delivery- mother immediately received information about the condition of the baby, of her condition and that she could immediately see her baby	4.34 (1.10)	82.2
	Health advices on caring for the newborn and breastfeeding	3.80 (1.40)	65.4
	Baby-Friendly Hospital program	3.82 (1.03)	63.3
	Procedures during admission and discharge from hospital	4.07 (1.21)	73.7
Communicational and interpersonal aspects of care	Kindness and achieved relationship of trust and understanding by obstetricians	4.34 (1.08)	81.9
	Kindness and achieved relationship of trust and understanding by midwives	4.34 (1.08)	81.3
	Kindness and achieved relationship of trust and understanding by neonatologists (pediatricians)	4.32 (1.08)	77.7
	Kindness and achieved relationship of trust and understanding by pediatric nurses	4.11 (1.14)	74.6

The scale of environmental factors generally showed the lowest satisfaction ratings, with nearly one-half of all mothers dissatisfied with hygiene and the sanitary facilities and quality of served meals. The highest average satisfaction score (4.43) referred to the overall participation of midwives during delivery. A high proportion of mothers were satisfied with the treatment and procedures during the preparation for childbirth (81%) and after delivery (82.2%). The satisfaction with communication and interpersonal aspects of care was very high, ranging from 74.6% of satisfied mothers with kindness and understanding of pediatric nurses to nearly 82% satisfaction towards obstetricians. Satisfaction with Baby-Friendly Hospital program was low, with average score of 3.82.

Based on univariate logistic regression analysis, the association between independent variables with maternal satisfaction revealed significant factors across different dimensions of satisfaction (Table 3). The variables significantly associated with all three dimensions of satisfaction were level of education, parity and the type of institution. Specifically, a lower level of education, multiparity, and hospitalization in general hospitals yielded increased maternal satisfaction. Normal vaginal delivery was associated with increased maternal satisfaction with environmental factors and communication and interpersonal aspects of care in comparison to the other two modes of delivery.

**Table 3:** Association of independent variables with mother’s satisfaction: univariate logistic regression analysis

**Variable**	**Satisfaction with environmental factors**	**Satisfaction with technical and professional aspects of care**	**Satisfaction with communicational and interpersonal aspects of care**
	**uOR (95% CI)**	**uOR (95% CI)**	**uOR (95% CI)**
Age			
≥25	0.922 (0.872-0.974)*	1.064 (0.997-1.135)	0.950 (0.904-0.999)*
<25	1.000	1.000	1.000
Education			
Higher level	0.646 (0.608-0.686)*	0.677 (0.629-0.729)*	0.703 (0.668-0.739)*
Lower level	1.000	1.000	1.000
Parity			
Multiparity	1.248 (1.187-1.313)*	1.350 (1.271-1.434)*	1.396 (1.334-1.460)*
Primiparity	1.000	1.000	1.000
Mode of delivery			
Vaginal with epidural anesthesia	0.887 (0.789-0.999)	0.851 (0.737-0.983)	0.808 (0.730-0.895)*
Caesarean	0.916 (0.863-0.973)*	0.986 (0.916-1.061)	0.941 (0.892-0.992) *
vaginal without	1.000	1.000	1.000
anesthesia			
Institution			
Primary health care	0.188 (0.161-0.220)*	0.227 (0.192-0.269)*	0.304 (0.248-0.372)*
Clinics and clinical	0.178 (0.152-0.209)*	0.174 (0.146-0.207)*	0.218 (0.178-0.267)*
centers			
General hospital	1.000	1.000	1.000
Mother informed of the patients’ rights			
Yes	2.904 (2.760-3.055)*	3.550 (3.338-3.776)*	2.768 (2.641-2.901)*
No or partly	1.000	1.000	1.000
Mother informed where she can file a complaint			
Yes	2.081 (1.977-2.191)*	2.607 (2.449-2.775)*	2.045 (1.955-2.139)*
No or partly	1.000	1.000	1.000
Mother informed through the Maternal Counseling Service			
Yes	2.432 (2.306-2.564)*	3.147 (2.948-3.360)*	2.472 (2.361-2.587)*
No or partly	1.000	1.000	1.000

uOR - unadjusted odds ratio.

*p < 0.05.

Multiple logistic regression analysis revealed that all three aspects of satisfaction were strongly associated with two variables (awareness of the patients’ rights and preparation for the pregnancy and delivery through the Maternal Counseling Service). The mothers informed of the patients’ rights and informed of pregnancy and delivery through the Maternal Counseling Service were more likely to be satisfied with environmental factors in the hospital, technical and professional aspects of care, and the communication and interpersonal aspects of care. Younger mothers and mothers with previous delivery experience were less concerned with the environmental conditions in the hospital wards (OR = 0.55, p = 0.006; and OR = 1.82, p = 0.004, respectively) Table 4.

**Table 4:** Final model of maternal satisfaction: multivariate logistic regression analysis

**Variable**	**Satisfaction with environmental factors**	**Satisfaction with technical and professional aspects of care**	**Satisfaction with communicational and interpersonal aspects of care**
	**aOR (95% CI)**	**aOR (95% CI)**	**aOR (95% CI)**
Age			
≥25	0.551 (0.360-0.843)*		
<25	1.000		
Parity			
Multiparity	1.821 (1.198-2.769)*		
Primiparity	1.000		
Mother informed of the patients’ rights			
Yes	2.761 (1.792-4.253)*	4.107 (2.380-7.087)*	4.302 (2.437-7.595)*
No or partly	1.000	1.000	1.000
Mother informed through the Maternal Counseling Service			
Yes	2.166 (1.408-3.333)*	4.254 (2.455-7.370)*	4.226 (2.395-7.459)*
No or partly	1.000	1.000	1.000

aOR (adjusted odds ratio): model includes the variables that were statistically significant in the bivariate univariate analysis.

*p < 0.05.

All three aspects of maternal satisfaction demonstrated a significant inter-correlation (p < 0.01) and correlation with the overall satisfaction score (Table 5). The highest value of the Pearson’s coefficient of correlation was observed between the overall satisfaction score and satisfaction with communication and interpersonal aspects of care.

**Table 5:** Correlations of overall satisfaction score with three aspects of maternal satisfaction

		**Overall satisfaction score**	**Satisfaction with environmental factors**	**Satisfaction with technical and professional aspects of care**	**Satisfaction with communicational and interpersonal aspects of care**
Overall satisfaction score	Pearson Correlation	1	.454^**^	.477^**^	.577^**^
	Sig. (2-tailed)		.000	.000	.000
	N	32562	32079	22535	31620
Satisfaction with environmental factors	Pearson Correlation		1	.465^**^	.343^**^
	Sig. (2-tailed)			.000	.000
	N		33593	22957	32203
Satisfaction with technical and professional aspects of care	Pearson Correlation			1	.491^**^
	Sig. (2-tailed)				.000
	N			23231	23020
Satisfaction with communicational and interpersonal aspects of care	Pearson Correlation				1
	Sig. (2-tailed)				
	N				32662

**Correlation is significant at the 0.01 level (2-tailed).

Less than one-third of the respondents filled in two open-ended questions in the questionnaire. Responding to the question about the aspects of care that they assessed as favorable, the answers were predominantly in the domain of communication and professionalism of the healthcare personnel. The greatest amount of praise was given to midwives. The thematic analysis of all answers about the aspects of hospital care that the women identified as being particularly unsatisfactory ascertained four different areas: hygiene in the wards, out-of-date equipment and hospital inventory, issues concerning time and duration of visits, and the possibility to get necessary information on breastfeeding and the care for the newborn.

During the phase of data collection and analysis, we became aware of certain limitations of our study instrument. In the future assessment of maternal satisfaction in our country this basic questionnaire should be amended with items which refer to the outcome of pregnancy and labor (health of mothers and newborns) and additional socio-economic variables, such as employment status, occupation, marital status, financial status and ethnicity. However, it should be noted that this was the first study of its kind in Serbia with a large number of participants. The results could be used as a basis for future comparisons and assessment of quality improvements of maternal care in public institutions.

## Discussion

The majority of published accounts about maternal satisfaction with obstetric care apply a cross-sectional study design using self-report questionnaires as a data collection instrument, while fewer studies have been based solely on qualitative methods, discussions of focus groups or interviews. A great number of studies also use the combination of quantitative and qualitative research methods. In our study, qualitative findings supported the results obtained by the quantitative method, emphasizing factors of critical importance for positive experience related to delivery in hospital wards. A recently published review of current measures of satisfaction with care during labor and birth emphasized the fact that despite the growing research interest, there are only a small number of validated instrument of satisfaction with care during labor and birth [18].

The differences in the methodological approaches of surveys refer to the timing of the research, location, and method of distributing of the questionnaires [19,20]. In the current study, midwives distributed the questionnaires to mothers, during the first home visit after delivery. Using a face-to–face recruitment and data collection procedure yielded a high response rate as a result of the convenience to the respondents. However, using personal contact and the presence of midwives is a well-known risk for response bias, and that fact should be considered when we discuss our results [21,22]. In addition, the timing of our survey (soon after delivery) may be a reason for the favorable satisfaction scores in many domains. As it has been demonstrated earlier in the literature [23], measuring satisfaction with respect to childbirth soon after delivery may be under the strong positive influence following the completion of labor and the joy of childbirth, while more negative aspects may take longer to integrate.

Our survey confirms the multidimensional nature of patient satisfaction assessment, and clearly allocates three important aspects of maternal satisfaction, as follows: environmental factors in hospitals; technical and professional aspects of care; and communication and interpersonal aspects of care. These three aspects of maternal satisfaction correspond to the findings of other studies [24]. The satisfaction with environmental factors in hospitals received the lowest median scores, and this result was confirmed and reinforced by the qualitative analysis of remarks which mainly referred to the hygiene and other factors from hospital environment. This was in accordance with the results of international studies, which have highlighted environmental factors as very significant predictors of maternal satisfaction [25,26]. According to our results and the results from an Australian study [27], younger and multiparous mothers expressed less concern regarding environmental factors. The positive attitude of multiparous mothers could reflect their more realistic expectations based on previous experiences with labor. Furthermore, these findings reinforce the suggestion from authors of a recent study in Sri Lanka [24], that more attention is needed to develop a better interpersonal relationship with women having their first delivery. Our findings are in contrast to the results from different satisfaction studies which have shown that women express fewer complaints and discontent with age [28].

The results on satisfaction with technical and professional aspects of care confirm the crucial role of professional competence of midwives during labor. Also, the communication and interpersonal aspects of care were assessed with very high median satisfaction scores, which indicated that our respondents were satisfied with the kindness and understanding of midwives and other health workers (obstetricians, neonatologists and nurses). Our findings, supported with the analysis of two open-ended questions, affirmed exceptional importance of a well-established relationship and good communication of healthcare personnel with mothers in hospitals, as highlighted in a study from Sweden. The Swedish study described women’s opinions about what is important to them during pregnancy and birth and stated that characteristics of midwives, such as being supportive, friendly, attentive, respectful and nonjudgmental, were the most desirable characteristics [29].

A very low satisfaction score with the WHO program (Baby-Friendly Hospital) indicated that the program needs to be improved in Serbian public hospitals and/or may also indicate that future mothers need to be more informed about the possibilities of the program before the hospitalization. This was not an unexpected result, although BFH was among national health priorities. However, the evaluation of the BFH initiative in Serbia for the period of 1995–2008 showed that there were many misperceptions of what is the BFH by many mothers, staff and managers as well as the wider community [30]. The initiative was moderately effective until about 2003 when support was reduced and activity curtailed, though low level activity continued in some areas due to the commitment of individuals. While some of the practices remain in place in some hospitals, the overall initiative with its assessment and monitoring of standards has not been sustained. The WHO/UNICEF global BFHI was updated in 2006–8 and the updated standards and supporting materials are not part of the activities in Serbia [30].

Our results also shed light on insufficient attention regarding the counseling about the pregnancy and delivery through the Maternal Counseling Service during the pregnancy, and revealed the unsatisfactory counseling of mothers about the care of newborn and breastfeeding.

According to the final model of multivariate analysis, two key factors in relation to higher maternal satisfaction in all three aspects are as follows: the awareness of the patients’ rights and the preparation for pregnancy and delivery through the Maternal Counseling Service. This finding is in accordance with the conclusions of other authors who have emphasized proper counseling and providing adequate information to all pregnant women as an important predictor of future maternal satisfaction with experience in the hospital during and after labor [31,32].

Awareness of patient rights and familiarity with all future procedures during delivery in the hospital give women a sense of control and participation in decision-making and consequently greater maternal satisfaction. Taking into consideration these facts, we are concerned with the result of nearly 60% of women who were not sufficiently informed about patients’ rights. The ongoing activities on patients’ rights protection in Serbia could lead towards improvement, but it takes time to become fully effective. Hence, in 2012, the Ministry of Health opened the draft of the first special Law on Patient Rights for public debate, which was enacted in June 2013 (Official gazette 45/13). In addition to the existing eleven rights (which already existed in Health Care Low), eight new rights were incorporated in the Law on Patient Rights. Among them are the right to quality health services, the right to a second opinion and the right to patient safety-all very relevant for the women delivered in public hospitals.

The evidences from similar studies suggest that patient satisfaction scores usually present a limited and optimistic picture. There is also a growing literature on the importance of measuring patient experiences of care, rather than exclusively satisfaction with care. Therefore, detailed assessment about specific aspects of patients’ experiences are likely to be more useful for monitoring the performance of various hospital departments and wards and could point to ways in which delivery of health care could be improved [33]. This could be the new course for our future studies on perinatal care in Serbian hospitals.

## Conclusions

Our study illuminated the importance of interpersonal aspects of care and education in order to reach greater maternal satisfaction with perinatal health care in hospitals. Apart from a clear need for improvement of the environmental conditions in hospitals, specifically hygiene, and the WHO program (Baby-Friendly Hospital), the main recommendations based on the results of this study were the enrollment of all pregnant women in antenatal education about pregnancy, birth and patients’ rights. Strengthening these factors will immensely affect women’s perception of quality of perinatal care in hospitals. The identified factors associated with maternal satisfaction provide valuable information for possible improvements of perinatal care in Serbian public hospitals.

## Endnote

^a^ Health care in Serbia is mainly financed by mandatory contributions to a social health insurance scheme. The National Health Insurance Fund (HIF) is responsible for financing the system and guarantees access to a relatively broad package of health services to the entire population. Principles like universal access and community-based services are health policy priorities. Primary health care is provided through the developed network of institutions at the municipality level (157 primary health care centers with the networks of health stations). Primary health care centers are providing outpatient care but according to the Regulation of health institutions network plan (Official gazette 42/06, 119/07, 85/09) small maternity wards with maximum 10 beds can be organized, if the distance from the nearest general hospital is more than 30 kilometers. General hospitals are the first referral level and the most universal part of hospital system with organized units for gynecology and obstetrics. Clinics and Institutes for gynecology and obstetrics belong to the health care at the tertiary level.

## Competing interests

The authors declare that they have no competing financial, professional or personal interests that might have influenced the performance or presentation of the work described in this manuscript.

## Authors’ contributions

BM designed the study and drafted the manuscript, MSM helped design the study and assisted with writing of the paper, VV performed statistical analysis, BDj assisted with advice interpreting results and discussion. All authors read and approved the final manuscript.

## Pre-publication history

The pre-publication history for this paper can be accessed here:

http://www.biomedcentral.com/1471-2393/14/14/prepub

## Supplementary Material

Additional file 1 Instrument for measuring maternal satisfaction with perinatal care in public hospitals. Click here for file

## References

[B1] Larrabee JH Bolden L Defining patient perceived quality of nursing care J Nurs Care Qual 2001 16 24 60 10.1097/00001786-200110000-00004 11668855

[B2] Worning AM Mainz J Klazinga N Gotric JK Johansen KS Policy and Quality Development for the Medical Profession J Dan Med Association 1992 145 49 3523 3533

[B3] Pittrof R Campbell OMR Filippi VGA What is quality in maternity care? An international perspective Acta Obstet Gynecol Scand 2002 81 277 283 10.1034/j.1600-0412.2002.810401.x 11952455

[B4] Sofaer S Firminger K Patient perceptions of the quality of health services Annu Rev Public Health 2005 26 513 559 10.1146/annurev.publhealth.25.050503.153958 15760300

[B5] Bramadat IJ Driedger M Satisfaction with childbirth: theories and methods of measurement Birth 1993 20 I 22 29 8503963 10.1111/j.1523-536x.1993.tb00175.x

[B6] Linder-Pelz S Toward a theory of patient satisfaction Soc Sci Med 1982 16 577 582 10.1016/0277-9536(82)90311-2 7100990

[B7] Williams B Patient satisfaction-A valid concept Soc Sci Med 1994 38 509 516 10.1016/0277-9536(94)90247-X 8184314

[B8] Hodnett ED Pain and women’s satisfaction with the experience of childbirth: a systematic review Am J Obstet Gynecol 2002 186 5 Suppl Nature 60 72 10.1067/mob.2002.121141 12011880

[B9] Dencker A Taft C Bergqvist L Lilja H Berg M Childbirth experience questionnaire (CEQ): development and evaluation of a multidimensional instrument BMC Pregnancy Childbirth 2010 10 81 10.1186/1471-2393-10-81 21143961 PMC3008689

[B10] Janzen JA Silvius J Jacobs S Laughter S Dalziel W Drummond N What is a health expectation? Developing a pragmatic conceptual model from psychological theory Health Expect 2006 9 37 48 10.1111/j.1369-7625.2006.00363.x 16436160 PMC5060332

[B11] Hulton LA Matthews Z Stones RW Applying a framework for assessing the quality of maternal health services in urban India Soc Sci Med 2007 64 2083 2095 10.1016/j.socscimed.2007.01.019 17374551

[B12] Hopkins A Costain D Measuring the Outcomes of Medical Care 1993 London: The Royal College of Physicians of London and King’s Fund Centre for Health Services Development

[B13] World Health Organization (WHO) Making Pregnancy Safer: The Critical Role of the Skilled Attendant: A Joint Statement by WHO,ICM,FIGO 2004 Geneva,Switzerland: WHO

[B14] Statistical office of the Republic of Serbia Demographic Yearbook 2009. Basic data and indicators of population changes Available at: http://webrzs.stat.gov.rs/WebSite/Public/PageView.aspx?pKey=162

[B15] Strategy for continous quality improvement and patient safetly. “Official Gazette-Republic of Serbia”, No 55/05

[B16] Humphrey and al The Caregiver Burden Questionnaire for Heart Failyre(CBH-HF): face and content validity Health Qual Life Outcomes 2013 11 84 10.1186/1477-7525-11-84 23706131 PMC3673843

[B17] Hsieh HF Shannon S Three approaches to qualitative content analysis Qual Health Res 2005 15 1277 1288 10.1177/1049732305276687 16204405

[B18] Sawyer A Ayers S Abbott J Gyte G Rabe H Duley L Measures of satisfaction with care during labour and birth: a comparative review BMC Pregnancy Childbirth 2013 13 108 10.1186/1471-2393-13-108 23656701 PMC3659073

[B19] Meakin R Weinman J The Medical Interview Satisfaction Scale (MISS-21) adapted for British general practice Pam Pract 2002 19 3 257 263 10.1093/fampra/19.3.257 11978716

[B20] Crow R Gage H Hampson S Hart J Kimber A Storey L Thomas H The measurement of satisfaction with healthcare: implications for practice from a systematic review of the literature Health Technol Assess 2002 6 32 1 244 12925269 10.3310/hta6320

[B21] Wensing M Elwyn G Metods for incorporating patients views in health care BMJ 2003 326 877 879 10.1136/bmj.326.7394.877 12702627 PMC1125777

[B22] Sitzia J Wood N Response rate in patient satisfaction research: an analysis of 210 published studies Int J Qual Health Care 1998 10 4 311 317 10.1093/intqhc/10.4.311 9835247

[B23] Waldenström U Why do some women change their opinion about childbirth over time? Birth 2004 31 2 102 107 10.1111/j.0730-7659.2004.00287.x 15153129

[B24] Upul S Dulitha NF Ishani R Factors determining client satisfaction with hospital-based perinatal care in Sri Lanka Trop Med Int Health 2006 11 9 1442 1451 10.1111/j.1365-3156.2006.01698.x 16930267

[B25] Waldenström U Rudman A Hildingsson I Intrapartum and postpartum care in Sweden: women’s opinions and risk factors for not being satisfied Acta Obstet Gynecol Scand 2006 85 5 551 560 10.1080/00016340500345378 16752233

[B26] Rudman A El-Khouri B Waldenström U Women’s satisfaction with intrapartum care - a pattern approach J Adv Nurs 2007 59 5 474 487 10.1111/j.1365-2648.2007.04323.x 17645495

[B27] Hauck Y Fenwick J Downie J Butt J The influence of childbirth expectations on Western Australian women’s perceptions of their birth experience Midwifery 2007 23 3 235 247 10.1016/j.midw.2006.02.002 17097202

[B28] Young G Meterko M Desai K Patient satisfaction with hospital care: effects of demographic and institutional characteristics Med Care 2000 38 325 334 10.1097/00005650-200003000-00009 10718357

[B29] Hildingsson I Thomas JE Women’s perspectives on maternity services in Sweden: processes, problems, and solutions J Midwifery Womens Health 2007 52 2 126 133 10.1016/j.jmwh.2006.10.023 17336818

[B30] UNICEF Evaluation of the Baby-Friendly Hospital Initiative in Serbia for the period 1995–2008 Available at: http://www.unicef.org/evaldatabase/index_67889.htm

[B31] Christiaens W Bracke P Assessment of social psychological determinants of satisfaction with childbirth in a cross-national perspective BMC Pregnancy Childbirth 2007 7 26 10.1186/1471-2393-7-26 17963491 PMC2200649

[B32] de Silva KS Dharmage SC Assessment of client satisfaction in a paediatric ward Ceylon Med J 1996 41 148 150 9141759

[B33] Jenkinson C Coulter A Bruster S Richards N Chandola T Patients’ experiences and satisfaction with health care: results of a questionnaire study of specific aspects of care Qual Saf Health Care 2002 11 4 335 339 S 10.1136/qhc.11.4.335 12468693 PMC1757991

