# Supplementary material for: Maternal satisfaction with organized perinatal care in Serbian public hospitals
Source: BMC Pregnancy Childbirth. 2014 Jan 13;14:14. doi: 10.1186/1471-2393-14-14 (PMC3916080; doi:10.1186/1471-2393-14-14)
Supplement: Additional file 1 — Instrument for measuring maternal satisfaction with perinatal care in public hospitals. [file 1471-2393-14-14-S1.doc]

**Instrument for measuring maternal satisfaction with perinatal care in public hospitals**

*We would like to find out how much you are satisfied with different aspects of care received during your stay in maternity ward. Please, mark the appropriate number that corresponds with your experience and satisfaction. Your opinion will be highly valued.*

| Aspects of care received during your stay in maternity ward | Fully dissatisfied | Somewhat dissatisfied | Neither satisfied nor dissatisfied | Somewhat satisfied | Fully satisfied |
| --- | --- | --- | --- | --- | --- |
| Cleanliness in the ward and the frequency of changing the nightgowns and bed sheets | 1 | 2 | 3 | 4 | 5 |
| Sanitary facilities (hygiene and equipment) | 1 | 2 | 3 | 4 | 5 |
| Room comfort in the ward | 1 | 2 | 3 | 4 | 5 |
| Quality of served food | 1 | 2 | 3 | 4 | 5 |
| Treatment during the preparation for childbirth | 1 | 2 | 3 | 4 | 5 |
| The extent to which obstetrician informed you with the plan of delivery, upcoming procedures and interventions and asked for your consent | 1 | 2 | 3 | 4 | 5 |
| The extent to which *obstetrician* devoted sufficient time to you, provided necessary information and answered your questions | 1 | 2 | 3 | 4 | 5 |
| The extent to which *midwife* devoted sufficient time, provided necessary information and answered your questions | 1 | 2 | 3 | 4 | 5 |
| The extent to which *neonatologist* devoted sufficient time, provided necessary information and answered your questions | 1 | 2 | 3 | 4 | 5 |
| The extent to which *pediatric nurse* devoted sufficient time, provided necessary information and answered your questions | 1 | 2 | 3 | 4 | 5 |
| Overall participation of midwives during delivery | 1 | 2 | 3 | 4 | 5 |
| Procedures after delivery- you immediately received information about the condition of your baby, of your condition and you could immediately see your baby | 1 | 2 | 3 | 4 | 5 |
| Health advices on newborn care and breastfeeding | 1 | 2 | 3 | 4 | 5 |
| Baby-Friendly Hospital program | 1 | 2 | 3 | 4 | 5 |
| Procedures during admission and discharge from hospital | 1 | 2 | 3 | 4 | 5 |
| Kindness and established trust and understanding by *obstetricians* | 1 | 2 | 3 | 4 | 5 |
| Kindness and established trust and understanding by *midwives* | 1 | 2 | 3 | 4 | 5 |
| Kindness and established trust and understanding by *neonatologists* (*pediatricians*) | 1 | 2 | 3 | 4 | 5 |
| Kindness and established trust and understanding by *pediatric nurses* | 1 | 2 | 3 | 4 | 5 |
